# Retinal venular constriction during hypercapnia in highlanders: The potential roles of oxygenation and occupational exposure

**DOI:** 10.1113/EP094340

**Published:** 2026-10-05

**Authors:** Faisal Ahsaan, Bakhtawar Latif, Aliza Akhtar

**Affiliations:** ^1^ Shaikh Khalifa Bin Zayed Al‐Nahyan Medical & Dental College Lahore Pakistan; ^2^ Quaid‐e‐Azam Medical College Bahawalpur Pakistan

**Keywords:** excessive erythrocytosis, high‐altitude hypoxia, hypercapnia, La Rinconada, occupational exposure, retinal vasoreactivity, retinal vessels

1

We read with interest the recent study by Paillisser et al. (2026), which examined retinal vascular phenotype and ocular vascular responses to hypercapnia in permanent residents of La Rinconada, Peru, at approximately 5100 m. The study addresses an unusual and clinically relevant population and provides useful information on how chronic exposure to extreme altitude and excessive erythrocytosis may influence the retinal circulation.

One finding, however, deserves further discussion. During the hypercapnic challenge, Central retinal venular equivalent (CRVE) decreased by 8.6 ± 22.0 µm (*P* = 0.02), while no clear arteriolar dilation was observed (Paillisser et al., 2026). The authors interpret these findings as evidence of altered retinal vascular reactivity after long‐term exposure to extreme altitude. An alternative explanation is that the hypercapnic stimulus changed not only CO_2_ but also oxygenation.

The protocol increased end‐tidal CO_2_ by approximately 10 mmHg but did not clamp end‐tidal O_2_ (Paillisser et al., 2026). In this setting, the rise in ventilation associated with hypercapnia could increase arterial and tissue oxygenation. The reported increase in $S_{pO_{2}}$ therefore indicates that the subjects became less hypoxemic during the challenge. This distinction is important. It would be preferable to describe this as a change in oxygenation or reoxygenation rather than hyperoxia, because the participants remained at high altitude and were not necessarily exposed to oxygen levels above normal.

Retinal vessels are responsive to changes in oxygen tension, and oxygen‐dependent vasoconstriction could therefore modify the response to CO_2_. In healthy subjects, CO_2_ breathing produces retinal arterial and venous dilation; Dorner et al. (2002) reported increases of 4.2% and 3.2%, respectively. Similarly, Venkataraman et al. (2008) demonstrated a 3.3% increase in retinal arteriolar diameter during experimentally controlled isoxic hypercapnia. The latter study is particularly relevant because O_2_ was maintained during the CO_2_ stimulus. These findings suggest that the absence of the expected dilation, and especially the reduction in CRVE, should be interpreted with caution when O_2_ was allowed to change during the challenge.

This does not establish that the change in oxygenation explains the entire finding. Rather, it raises the possibility that the retinal response reflected the combined effects of CO_2_ and O_2_ rather than an isolated response to hypercapnia. Future studies using prospective end‐tidal O_2_ and CO_2_ control would help separate these effects.

A second issue concerns occupational exposure. The study population was drawn from the mining community of La Rinconada, where many residents work in or around artisanal gold mining (Paillisser et al., 2026). This is relevant because the authors' related work in the same setting has already identified mining activity as a possible source of confounding, noting that silicosis and mercury contamination could not be excluded (Savina et al., 2024). Mercury exposure is also biologically relevant because experimental and review literature has linked mercury exposure with oxidative stress and impaired nitric oxide‐dependent vascular function (Martins et al., 2021). These observations do not show that mining exposure caused the retinal findings in the present study, but they support its consideration as an additional factor when interpreting vascular reactivity in this population.

The retinal phenotype observed by Paillisser et al. is therefore unlikely to have a single explanation. Chronic hypoxia and excessive erythrocytosis may contribute, but the acute change in oxygenation during hypercapnia and the occupational environment may also influence vascular responses. This distinction is important when attempting to infer long‐term vascular adaptation from an acute physiological challenge.

We believe that future studies combining isoxic hypercapnia with careful measurement of end‐tidal gases, together with non‐mining comparison groups or direct assessment of occupational exposure, would provide a clearer picture of retinal vascular adaptation at extreme altitude.

## AUTHOR CONTRIBUTIONS

All authors satisfy the ICMJE criteria for authorship. Faisal Ahsaan: Conceptualization, Writing—original draft, Writing—review & editing. Bakhtawar Latif: Conceptualization, Writing—original draft, Writing—review & editing. Aliza Akhtar: Conceptualization, Writing—original draft, Writing—review & editing. All authors have read and approved the final version of this manuscript and agree to be accountable for all aspects of the work in ensuring that questions related to the accuracy or integrity of any part of the work are appropriately investigated and resolved. All persons designated as authors qualify for authorship, and all those who qualify for authorship are listed.

## CONFLICT OF INTEREST

None declared.

## FUNDING INFORMATION

None.

## GENERATIVE AI STATEMENT

No generative AI tools were used in the preparation of this manuscript.
